# Serum Exosomal miRNAs Are Associated with Active Pulmonary Tuberculosis

**DOI:** 10.1155/2019/1907426

**Published:** 2019-02-11

**Authors:** Shamila D. Alipoor, Payam Tabarsi, Mohammad Varahram, Mehrnaz Movassaghi, Mehdi Kazempour Dizaji, Gert Folkerts, Johan Garssen, Ian M. Adcock, Esmaeil Mortaz

**Affiliations:** ^1^Institute of Medical Biotechnology, Molecular Medicine Department, National Institute of Genetic Engineering and Biotechnology (NIGEB), Tehran, Iran; ^2^Department of Biotechnology, School of Advanced Technologies in Medicine, Shahid Beheshti University of Medical Sciences, Tehran, Iran; ^3^Clinical Tuberculosis and Epidemiology Research Center, National Research Institute of Tuberculosis and Lung Diseases (NRITLD), Shahid Beheshti University of Medical Sciences, Tehran, Iran; ^4^Mycobacteriology Research Center, National Research Institute of Tuberculosis and Lung Diseases (NRITLD), Masih Daneshvari Hospital, Shahid Beheshti University of Medical Sciences, Tehran, Iran; ^5^Division of Pharmacology, Utrecht Institute for Pharmaceutical Sciences, Faculty of Science, Utrecht University, Utrecht, Netherlands; ^6^Nutricia Research Centre for Specialized Nutrition, Utrecht, Netherlands; ^7^Airways Disease Section, National Heart & Lung Institute, Imperial College London, London, UK; ^8^Priority Research Centre for Healthy Lungs, Hunter Medical Research Institute, The University of Newcastle, Newcastle, NSW, Australia

## Abstract

**Introduction:**

Tuberculosis (TB) remains a major threat to human health. Due to the limited accuracy of the current TB diagnostic tests, it is critical to determine novel biomarkers for this disease. Circulating exosomes have been used as diagnostic biomarkers in various diseases.

**Objective of the Study:**

In this pilot study, we examined the expression of miRNAs as biomarker candidates for the diagnosis of TB infection.

**Methods:**

Serum-derived exosomes were isolated from TB patients and matched control subjects. The expression of miR-484, miR-425, and miR-96 was examined by RT-PCR methods.

**Results:**

The expression of miR-484, miR-425, and miR-96 were significantly increased in serum of TB patients which correlated with the TB infection level. A receiver operating characteristic (ROC) curve analysis showed the diagnostic potency of each individual serum exosomal miRNA with an area under the curve (AUC) = 0.72 for miR-484 (*p* < 0.05), 0.66 for miR-425 (*p* < 0.05), and 0.62 for miR-96 (*p* < 0.05).

**Conclusion:**

These results demonstrate that exosomal miRNAs have diagnostic potential in active tuberculosis. The diagnostic power may be improved when combined with conventional diagnostic markers.

## 1. Introduction

Tuberculosis (TB) is the most common cause of death from infectious diseases. Despite global advances in health and medicine, tuberculosis remains an important global health challenge [1]. The WHO reported 11 million new TB cases and 1.4 million TB deaths in 2016 [2].

Because of the limitations in the current TB diagnostic methods and the lack of an optimal method, clinicians are still faced with the challenge of early diagnosis [3]. Since early detection of TB has an important role in controlling the disease and preventing infections from spreading, the introduction of novel biomarkers will be extremely valuable [4].

Exosomes are novel diagnostic biomarkers used in a wide range of diseases such as cancers and infectious diseases [5, 6]. Exosomes are 30–100 nm vesicles secreted from most cell types and can be found in nearly all human biofluids [7]. Exosomes have an important role in cell to cell communication as they shuttle biological information in the form of different molecules including microRNAs (miRNAs) between cells [8]. Indeed, exosomal contents have been identified as signatures of various diseases including Alzheimer's disease [9] and various cancers including myeloid leukemia (AML) [10].

miRNAs are small 18–22 nucleotide noncoding RNAs that act in the posttranscriptional regulation of gene expression. MicroRNAs are the key players of most biological functions, and their dysregulation can lead to several pathological outcomes [11]. Importantly, functional miRNAs encapsulated within exosomes can be delivered to recipient cells and induce specific modulation of their transcriptomes [8]. In addition, miRNAs are implicated in regulating inflammatory processes after Mtb infection. Mtb infection leads to a variety of host physiological responses including host immune and metabolic repatterning [12] which enables Mtb to maintain their nutritional needs and energy requirements and promote their intracellular survival [13]. This process involves the modulation of host miRNAs that control the regulatory networks associated with carbon, nitrogen, and lipid metabolism of the infected cells [14].

In a previous study, we observed that infection of human monocyte-derived macrophages (MDM) with Mycobacterium bovis bacillus Calmette-Guerin (BCG) induced the secretion of a specific set of exosomal miRNAs that were involved in modulating key host metabolic and energy production pathways as well regulating immunological and cell signaling events [15]. We hypothesized, therefore, that exosomal miRNAs released from Mtb-infected cells might have potential as diagnostic biomarkers of active disease. In a small pilot study, we examined the expression of the top 3 miRNA hits (miR-484, miR-425, and miR-96-3P) that modulate these critical pathways in serum exosomes from patients with TB to determine their potential as a biomarker for TB diagnosis and/or activation status.

## 2. Materials and Methods

### 2.1. Patients and Samples

25 patients newly diagnosed with TB aged 18–65 years were recruited at the Masih Daneshvari Hospital between April 2015 and September 2016. The criteria for enrollment were clinical and radiological findings indicating pulmonary TB including mycobacterial culture or a positive bronchial washing specimen obtained at bronchoscopy (Table 1). 25 healthy age- and gender-matched controls with a negative history of TB disease were also recruited. All the control subjects were tested for prior exposure to TB using QuantiFERON-TB Gold (QFT®) tests and were negative in result. Sputum smear tests were performed and graded according to infectivity. Patients were divided into 4 groups based on smear test positivity.

Blood samples were collected from all subjects and then centrifuged at 1500 × g for 15 min at 4°C. Sera were isolated and stored at −80°C until use. The Ethics Committee of Dr. Masih Daneshvari Hospital, Tehran, Iran, approved the study. Informed consent was obtained from all participants and/or their legal guardian/s.

### 2.2. Isolation and Characterization of Serum-Derived Exosomes

Serum exosomes were isolated using the total exosome isolation (TEI) reagent (Invitrogen, Thermo Fisher Scientific Corporation, Waltham, MA, USA). Briefly, sera were centrifuged for 30 min at 300 × g and filtered through a 0.22 *μ*m filter (Merck Millipore, Billerica, MA, USA). TEI solution was added at a 1 : 5 ratio, and samples were incubated at 4°C for 30 min before centrifugation at 10000 × g for 1 h. The exosomal pellet was resuspended in 1 ml of PBS (Sigma, Munich, Germany) before storing at −70°C until use. Isolated exosomes were confirmed by electron microscopy (Carl Zeiss NTS, Oberkochen, Germany) and flow cytometry (FACS Calibur, BD, USA).

For flow cytometric analysis, 0.45 mg of exosomes was coupled with 4 *μ*m of aldehyde/sulfate latex beads (Thermo Fisher) overnight at 4°C on a rotator. The remaining binding sites were blocked by incubation with 100 mM glycine (Sigma) for 30 min and then stained with CD81 antibody or an isotype control (BD Biosciences, San Jose, CA, USA). Data were collected by flow cytometry (FACS Calibur) and analyzed using Flow software (BD Biosciences).

### 2.3. Exosomal RNA Extraction and cDNA Synthesis

To eliminate nonexosomal RNAs, exosomes were first treated with RNase A (5 *μ*g/*μ*l Fermentase, Thermo Fisher, USA) for 90 min at 37°C. Total RNA was extracted from the exosomes with TRIzol (Invitrogen) according to the manufacturer's protocol. The concentration and purity of the isolated exosomal RNA were determined using a NanoDrop 2000 spectrophotometer (NanoDrop Technologies Inc., Wilmington, DE, USA). The quality and size of extracted RNA were determined using capillary electrophoresis (Agilent 2100 Bioanalyzer, Agilent Technologies, Foster City, CA, USA). Extracted RNA (20 ng) was reverse transcribed using the miRCURY LNA Universal RT microRNA cDNA Synthesis Kit (Exiqon, Vedbæk, Denmark) according to the manufacturer's instructions.

### 2.4. Real-Time Quantitative PCR and miRNA Quantification

Real-time PCR was performed using the ExiLENT SYBR® Green Master Mix kit (Exiqon) for miR-96, miR-484, and miR-425 according to the manufacturer's instructions. Locked nucleic acid (LNA) primers (Exiqon) were used in all the experiments. cDNA was diluted 10x and added to the PCR reactions. A two-step real-time PCR protocol was performed using an initial denaturation step at 95°C for 10 min, 45 amplification cycles including a denaturation step (10 s at 95°C), and an annealing step (60 s at 60°C). A melting curve was determined for each reaction to confirm the precision of the reactions. Expression levels of all the miRNAs were normalized to the level of U6 as a control using the 2^–ΔΔCt^ method.

### 2.5. miRNA Target Genes and Enrichment Analysis

MicroRNA target genes were determined using miRTarBase [16] and microT-CDS algorithms [17]. An enrichment analysis was performed using Enrichr [18] and the KEGG pathway database [19].

### 2.6. Statistical Analysis

All experiments were performed at least 3 times and analyzed for significance (*p* < 0.05) using analysis of variance (ANOVA) and a postanalysis Student's *t*-test. Testing of serum exosomal miRNAs as TB biomarkers was performed using the sensitivity, specificity, and area under the receiver operating characteristic (ROC) curve. The area under the curve (AUC) was resolved with a 95% confidence interval (CI). ROC analyses were performed using SPSS (v.16).

## 3. Results

### 3.1. Characterization of Serum Exosomes and Exosomal miRNAs

**Figure 1:**
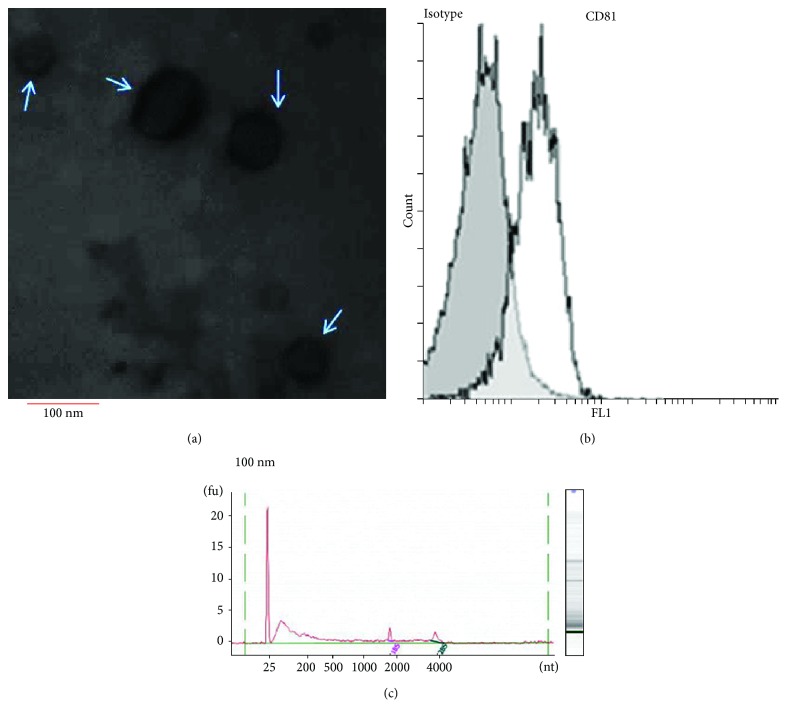
Characterization of the serum exosomes. (a) Transmission electron microscopy showing that serum exosomes are spherical particles with an average size of 70 nm. The bar represents 100 nm. (b) Detection of exosomal CD81 surface markers using flow cytometry. MFI (mean fluorescence intensity) represents the expression of CD81 on the surface of exosomes. The results are representative of three independent experiments. (c) Exosomal RNA analysis by Agilent Bioanalyzer indicated a population of 18–28 nt without prominent ribosomal RNA peaks. Results are representative of three independent experiments.

Exosomes isolated from the serum of healthy and control subjects were morphologically confirmed by transmission electron microscopy (TEM) (Figure 1(a)). Serum exosomes contain spherical particles with an average size of 70 nm. These exosomes expressed high levels of the exosomal marker protein CD81 as determined by flow cytometry (Figure 1(b)).

MicroRNA yields from 500 *μ*l serum ranged from 12 to 18 ng as determined by a NanoDrop spectrophotometer. Analysis using an Agilent Bioanalyzer indicated that the miRNAs were 18–28 nt in length and contained no prominent ribosomal RNA peaks confirming the presence of a small RNA population in the exosomes (Figure 1(c)).

### 3.2. The Exosomal Presence of miR-484, miR-425, and miR-96 Increases in TB Patients Compared to Controls

**Figure 2:**
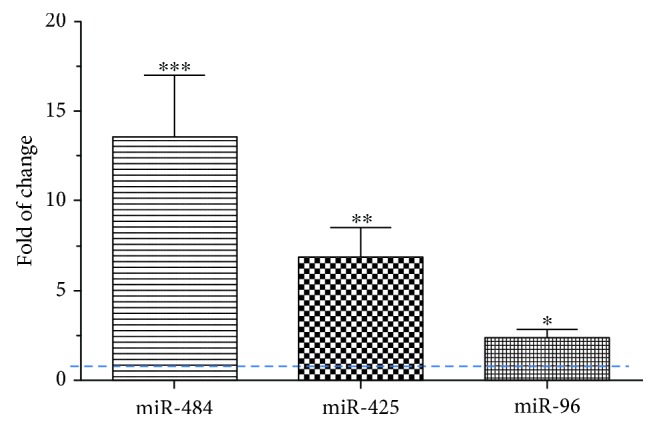
The relative expression of exosomal miR-484, miR-425, and miR-96-3-p in TB patients compared to that in control subjects. Real-time PCR of exosomal miR-484, miR-425, and miR-96 indicated upregulation in TB patients compared to control subjects (^∗^*p* < 0.05 and ^∗∗^*p* < 0.01 significantly different compared to control). Data represent mean ± SEM of data from 25 patients in each group. Each sample was analyzed twice in triplicate to give a single value for each subject. The relative value in control subjects is defined by the dotted blue line.

The expression of the nuclear RNA U6 was used as an endogenous control for all subsequent experiments. All analyses were performed in triplicate on 2 independent samples from each subject. The relative expression of these miRNAs compared to U6 was consistent in healthy control and was used as a comparator value for samples from patients with disease. After normalization to U6, a statistically significant upregulation of miR-484 (13.55 ± 3.44 − fold increase, *p* ≤ 0.01), miR-425 (6.84 ± 1.7 − fold increase, *p* ≤ 0.01), and miR-96 (2.37 ± 0.53 − fold increase, *p* ≤ 0.05) was demonstrated in TB patients in comparison to healthy controls (Figure 2).

### 3.3. Correlation between the Exosomal Level of miRNAs and the Grade of Smear Positivity

**Figure 3:**
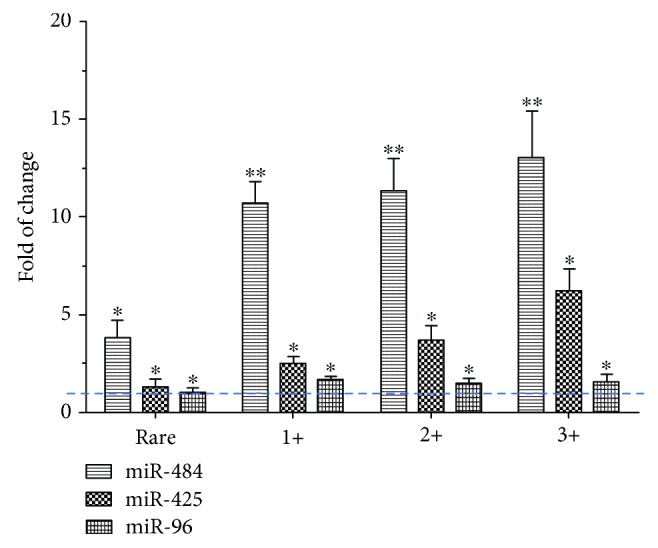
Correlation between the exosomal level of miRNAs and the grade of smear positivity. Real-time PCR showed increased exosomal miRNA expression with increasing infection rates in comparison with healthy controls. Data represent mean ± SEM from 25 patients of 2 independent analyses each repeated in triplicate for each subject. ^∗^*p* < 0.05 and ^∗∗^*p* < 0.01 significantly different compared to control. The relative value in control subjects is defined by the dotted blue line.

We further examined the relationship between the expression level of exosomal miRNAs and the grade of smear positivity. There was a trend towards increased levels of the 3 serum exosomal miRNAs with increasing numbers of bacteria in the sputum (Figure 3). This was most evident with miR-484 whose expression significantly increased in comparison to control subjects in rare positive patients (3.56 ± 0.88 − fold increase), 1+ subjects (10.29 ± 1.10 − fold increase), 2+ subjects (11.58 ± 1.70 − fold increase), and 3+ subjects (13.11 ± 2.39 − fold increase).

**Figure 4:**
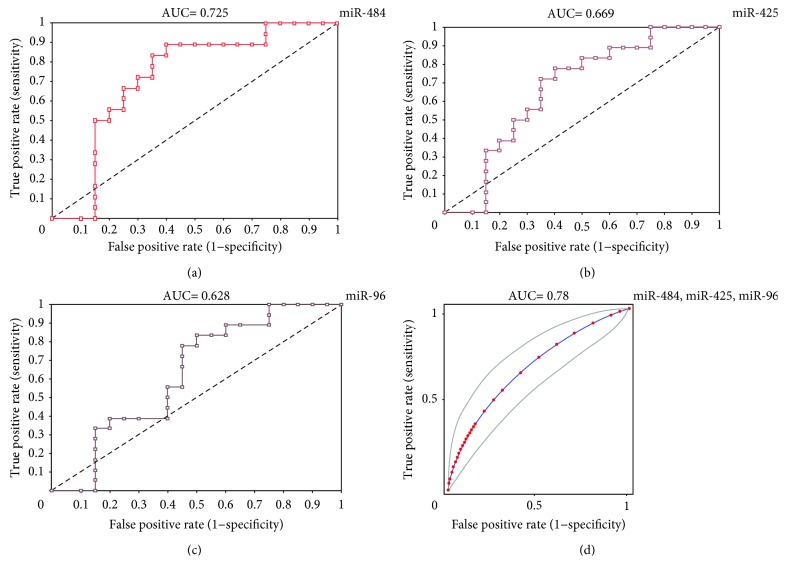
Diagnostic power of exosomal miR-484 (a), miR-425 (b), and miR-96 (c) determined by ROC curve analysis. The results show the area under the ROC curve (AUC) for the sensitivity and specificity of each miRNA. The improved AUC (95% CI) for the combination of all miRNAs is shown in (d).

A similar significant and graded increase was seen with miR-425 (1.29 ± 0.42-, 2.56 ± 0.36-, 3.76 ± 0.74-, and 6.05 ± 1.17-fold increases in rare positive, 1+, 2+, and 3+ subjects, respectively). Although miR-96 was increased in 1+ patients compared to rare positive and control subjects, the expression was not enhanced with an increasing grade of smear positivity (0.97 ± 0.22-, 1.53 ± 0.18-, 1.51 ± 0.24-, and 1.41 ± 0.34-fold increases in rare positive, 1+, 2+, and 3+ patients, respectively (Figure 3)). We also evaluate whether these putative serum exosomal miRNAs had potential to be considered as biomarkers for TB by performing ROC curve analyses. Each individual miRNA alone could differentiate TB infection from healthy controls with AUC = 0.72 (95% CI: 0.67–0.77) for miR-484 (*p* < 0.05), 0.66 (95% CI: 0.58–0.75) for miR-425 (*p* < 0.05), and 0.62 (95% CI: 0.53–0.71) for miR-96 (*p* < 0.05) (Figure 4).

Combinations of miRNAs generally improved the AUC (Table 2, Figure 4). A panel of miR-484, miR-425, and miR-96 gave an AUC = 0.78 (95% CI: 0.73–0.83) (*p* < 0.05) whilst combining miR-484 and miR-425 gave an AUC = 0.71 (95% CI: 0.69–0.74). Combining miR-96 with either miR-484 or miR-425 gave an AUC ≤ 0.6 (Table 2, Figure 4).

### 3.4. Enrichment Analysis of the Candidate miRNA Target Genes

To further survey the interactions between the genes targeted by the dysregulated miRNAs, an enrichment analysis was performed. Pathway analysis in KEGG, Reactome, and wiki-pathways using Enrichr revealed that the studied target genes were mostly involved in signaling pathways, metabolism, and immunological pathways (Table 3).

## 4. Discussion

We show in this pilot study that there is increased expression of miR-484, miR-425, and miR-96 in serum exosomes of patients with TB. Subgroup analysis showed that the level of miRNAs was associated with bacterial burden. Using ROC curve analysis, we show that the individual serum exosomal miRNAs have a fair predictive value for TB but this predictive value increases to good depending upon the increase of degree of smear positivity using miR-484 and miR-425 and by combining miRNA expression levels.

BCG infection of human macrophages *in vitro* induces the exosomal release of 11 miRNAs which are involved in the regulation of several host key pathways including metabolic pathways, cell signaling pathways, and infection-related pathways which are involved in energy production and intracellular bacterial survival [15]. Since the similarity between the miRNA profiles induced by BCG and Mtb has been previously demonstrated [20], we selected a subgroup of these differentially expressed miRNAs (miR-484, miR-425, and miR-96) to assess whether these serum exosomal miRNAs could act as putative candidate biomarkers in TB patients.

Relative miRNA profiles have been examined in TB patients from a number of different compartments including serum-free miRNAs and macrophages [21]. In addition, the exosomal protein content has been assessed as a potential biomarker for TB [22]. Initial array-based profiling of serum circulating miRNAs in response to Mtb infections demonstrated that 92 serum circulating miRNAs were significantly different in TB patients compared to healthy controls [23, 24]. Yi et al. in 2011, again using an array-based approach, described a cluster of 95 miRNAs which were expressed differentially in sputum from TB patients in comparison with control subjects [23].

Generally, previous studies reporting on miRNA expression profiles in TB have not been consistent [24]. A possible reason for these inconsistent results may reflect the focus on free rather than on exosomal miRNAs. Free miRNAs in human serum or sputum are exposed to nucleases and other degrading conditions which may affect the results obtained [25]. In addition, the encapsulation of miRNAs in exosomes allowed the increased stability of the RNA making the results less dependent on sample storage conditions [7]. Thus, exosome-enclosed miRNAs are now being considered as potential biomarkers in many diseases such as several cancers [7].

The miRNAs studied here have important roles in cell metabolism. miR-425 controls several metabolic pathways and is linked to metabolic disorders [26] whilst miR-484 targets the mitochondrial fission protein 1 (Fis 1) to modulate intermediate metabolic pathways [27]. In addition, miR-425 and miR-96 are associated with insulin resistance [28, 29]. miR-96 can regulate insulin secretion by increasing the level of granuphiline, an inhibitor of insulin exocytosis [30], and generally regulates the expression of multiple genes that fine tune insulin release. Physiologically, miR-96 is strongly induced by fatty acids and downregulates the expression of the insulin receptor (INSR) and the insulin receptor substrate 1 (IRS-1) to induce a failure in insulin signaling and glycogen synthesis in hepatocytes [29].

In support of this, Mtb-infected macrophages show altered levels of intracellular glucose, glycogen, NAD and NADP, and lactate reflecting a disturbed sugar flux [13]. Mtb infection also results in a pentose-phosphate shunt and glucose uptake which leads to an increased aerobic glycolysis [13]. Finally, Mtb-infected lung tissue showed significant changes in metabolomics profiling using H-NMR [31].

Serum exosomes from TB patients contained increased levels of miRNAs associated with dysregulated metabolism indicating that they may reflect the metabolic reprogramming which occurs in infected cells to favour Mtb survival. It may, therefore, be possible to use these exosomal dysregulated miRNAs as biomarkers for TB therapy monitoring as well as for diagnosis of active disease. The diagnostic efficacy of the 3 miRNAs tested individually in this pilot study using ROC analysis was fair (0.7–0.8) and was improved by using combined miRNAs (>0.85).

There are some limitations to our study. Although we observed an association between miR-484, miR-425, and miR-96 and the smear test positivity grade, a known measure of disease severity and infectivity, we did not have a comparison group of latent infected patients or patients with a different infection. Some studies have shown that patients with cavitating pulmonary TB have a higher bacterial burden in their sputum [32, 33], and other studies indicate that sputum smear test positivity was linked to the area of alveolar infiltration [34]. In addition, other scoring systems for TB severity also report an association of disease severity with acid-fast bacilli (AFB) results [35]. Subgroup analysis revealed that the level of exosomal miRNAs was significantly increased in TB patients with a higher degree of smear positivity, and the ROC curve analysis demonstrated that the diagnostic performance was captured better in patients with a higher bacterial burden. However, our study excluded patients with other infections or known infection with other mycobacterial infections and also those with latent TB. Subsequent studies should include these groups as controls.

We aimed to show the possible association of exosomal miRNAs with active tuberculosis and, based on our result, these exosomal miRNAs may be considered to be evaluated as potential biomarkers in future studies. The effectiveness of these miRNAs as biomarkers may be improved by combination with conventional biomarkers. Further studies are needed in large multicenter longitudinal cohorts of patients with active and latent tuberculosis, in TB patients before and after treatment, and in infectious disease control subjects to confirm their true value as a possible diagnostic biomarker for TB infection/latency or for active TB. The diagnostic accuracy of the combined markers is generally higher than that of a single one; combining the serum exosomal miRNA expression data with current testing strategies may improve the accuracy of early active TB diagnosis and may be a convenient and cost-effective method for screening TB infection. These data highlight that exosomal miRNAs may be considered as possible biomarkers for active TB.

## Figures and Tables

**Table 1:** Clinical characteristics of the patients with active TB (*n* = 25).

Characteristic	Value
*Age (years, range)*	41 (15–65)
*Gender (men, women)*	12, 13
*History*	
Diabetes mellitus	0
Others (malignancy, HIV, or other infectious diseases)	0
*Clinical presentation*	
Cough/sputum	20
Fever	5
Hemoptysis	0
*Diagnosis*	
Culture, AFB, and PCR	25

AFB: acid-fast bacillus; DM: diabetes mellitus; PCR: polymerase chain reaction.

**Table 2:** Predictive values from AUC data from ROC curves for serum exosomal miR-484, miR-425, and miR-96 individually and in combination in TB.

miRNA	AUC
miR-484	0.72
miR-425	0.66
miR-96	0.62
miR-484, miR-425	0.71
miR-425, miR-96	0.64
miR-484, miR-96	0.68
miR-484, miR-425, and miR-96	0.78

**Table 3:** Target genes and pathways of miR-484, miR-425, and miR-96.

Cluster	Node ID in cluster	Pathways	
		Pathway name and function	Mechanism
1	VEGFD, VEGFB, VEGFA, PDGFB, PDGFA, CUL7, CUL5, CUL4B, CUL4A, CUL3, CUL2, CUL1, CACUL1	Endocytosis Bacterial invasion of epithelial cells EGFR receptor signaling Golgi-associated vesicle biogenesis Signal transduction Clathrin-derived vesicle budding	Mycobacterium can exploit the formation of new blood vessels to facilitate its dissemination via VEGF-related signaling pathways [36]. Upregulation of PEGF secreted by alveolar macrophages upon mycobacterium infection and in company with the EGFR signaling pathway manipulates host lipid metabolism [37]. Cullins (CUL) provide a scaffold for ubiquitin ligases (E3) involved in protein catabolism [38].

2	TGFBR1, TCF7, TCF4, PRKG1, MAN1A2, GNAI1, EDEM1, CTNNB1	TGF-*β* receptor signaling pathways Adherents junction Focal adhesion Regulation of lipid metabolism Fatty acid, triacylglycerol, and ketone body metabolism	Regulation of the immune system Mycobacterium dissemination Metabolism and catabolism

3	SOS1, PANK3, IQGAP1, PRKCA, INPP4B, PIKFYVE, MAN2A1	N-Glycan biosynthesis Proteasome degradation Asparagine N-linked glycosylation T-Cell receptor and costimulatory signaling ErbB signaling pathway Downstream signaling of activated FGFRs VEGFA-VEGFR2 pathway	Metabolism and catabolism Immune system regulation New vessel formation

4	UBE2W, SMURF2, RYR2, PTEN, PLCZ1, MAN1A1, GRM1, GNAQ	Ubiquitination & proteasome degradation Calcium signaling pathway Class I MHC-mediated antigen processing & presentation Gap junction Inositol phosphate metabolism Insulin secretion Phosphatidylinositol signaling system Phospholipid metabolism	Adaptive immune system Antigen processing: Metabolism and catabolism

5	VEGFC, ST6GAL1, PRKCB, PLN, PIK3CD, PDGFD, ORAI3, OCRL, MAN2C1, MAN2B1, MAN2A2, LEF1, HERC3, EDEM2	Calcium signaling pathway Focal adhesion Asparagine N-linked glycosylation Beta-catenin-independent WNT signaling Downstream signaling events of B cell receptor (BCR) IL-3 signaling pathway N-glycan antennae elongation in the medial/trans-Golg Phosphatidylinositol signaling system PI metabolism Rap1 signaling pathway Ras signaling pathway Signaling by receptor tyrosine kinases	Metabolism and catabolism Modulation of signaling pathways Cytokines modulation

VEGF: vascular endothelial growth factor; CUL: cullin; TGFBR1: transforming growth factor beta receptor I; TCF7: transcription factor 7; TCF4: transcription factor 4; PRKG1: protein kinase cGMP-dependent 1; MAN1A2: mannosidase alpha class 1A member 2; GNAI1: guanine nucleotide-binding protein G(i); EDEM1: ER degradation enhancing alpha-mannosidase-like protein 1; CTNNB1: catenin beta1.

## Data Availability

No data are available on-line. Contact EM for further information if necessary.
